# Spatial prediction of canine visceral leishmaniasis in an endemic urban area of Brazil

**DOI:** 10.1371/journal.pone.0330730

**Published:** 2025-08-29

**Authors:** Patricia Sayuri Silvestre Matsumoto, Juliana Mariotti Guerra, Roberto Mitsuyoshi Hiramoto, Helena Hilomi Taniguchi, Denise Maria Bussoni Bertollo, Mariana Cortês Boité, Khan Rahaman, Mathew Novak, Bruno Cogliati, Elisa Cupolillo, Raul Borges Guimarães, José Eduardo Tolezano, Archie Campbell Adair Clements

**Affiliations:** 1 Department of Geography and Environmental Studies, Saint Mary’s University (SMU), Halifax, Nova Scotia, Canada; 2 Faculty of Health Sciences, Curtin University, Perth, Western Australia, Australia; 3 Pathology Center, Adolfo Lutz Institute (IAL), São Paulo, São Paulo, Brazil; 4 Department of Pathology, School of Veterinary Medicine and Animal Sciences, University of São Paulo (USP), São Paulo, São Paulo, Brazil; 5 Parasitology and Mycology Center, Adolfo Lutz Institute (IAL), São Paulo, São Paulo, Brazil; 6 Regional Laboratory Center, Adolfo Lutz Institute (IAL), São José do Rio Preto, São Paulo, Brazil; 7 Oswaldo Cruz Foundation (FIOCRUZ), Rio de Janeiro, Rio de Janeiro, Brazil; 8 Department of Geography, São Paulo State University (UNESP), School of Technology and Sciences, Presidente Prudente, São Paulo, Brazil; 9 School of Biological Sciences, Queen’s University Belfast, Belfast, United Kingdom; UFSJ: Universidade Federal de Sao Joao del-Rei, BRAZIL

## Abstract

Canine visceral leishmaniasis (CVL) is a widespread zoonotic disease in Brazil. This study aimed to identify and predict spatial patterns of CVL in an endemic city, Votuporanga, and examine disease associations with key environmental and anthropogenic factors at a fine spatial scale. First, we estimated the spatial clustering of CVL cases relative to non-cases from 8,146 dogs. Second, we assessed CVL density using a Kernel density ratio map. Third, we analyzed associations between disease occurrence and selected variables derived from the Normalized Difference Vegetation Index (NDVI), number of buildings, building area, and street density using binary logistic regression models. Finally, we predicted the spatial odds of CVL using a Generalized Additive Model (GAM) that incorporated the significant covariates. Our results revealed significant clustering of cases up to a range of 1.7 km. Mean NDVI, street density, and sparse vegetation were statistically significant, increasing the odds of CVL by 431%, 109%, and 100%, respectively, per unit change. The predictive performance of the GAM, evaluated through cross-validation, indicated that the model incorporating mean NDVI achieved the best fit, with an area under the receiver operating characteristic (ROC) curve of 0.74 (CI 0.72–0.76). Our findings demonstrate that CVL is widespread across the city, predominantly in urban fringe areas, with nearly 45% of the city classified as having increased odds of CVL (>1). In contrast, the downtown area exhibited lower odds of disease. Furthermore, we identified distinct parasite genotypes across the city, primarily in areas with higher disease odds. Altogether, our results highlight how biological and environmental data can be integrated into mapping to enhance the understanding of the spatial dynamics of disease transmission in urban areas.

## 1. Introduction

Visceral leishmaniasis (VL) is a vector-borne disease widely dispersed globally, except in Oceania [1]. It is a systemic disease that mainly affects vulnerable age groups such as children under five years of age, people aged over 50 and adults associated with comorbidities or immunosuppressive conditions such as HIV-AIDS [2]. As a systemic disease, VL can have severe and potentially fatal consequences if left untreated [1,2]. Globally, Sudan, Ethiopia, Brazil, Kenya and South Sudan were responsible for 72% of the reported cases in 2023 [3]. In the Americas, a total of 73,092 new cases of VL were recorded from 2001 to 2023, with an average of 3,178 cases per year [4]. In 2023, 91% of the cases occurred in Brazil [4].

The State of São Paulo is one of the most VL heavily affected regions in Brazil. The northwest part of the State is endemic for VL, and the first canine and human cases were reported in 1998 and 1999, respectively, in Araçatuba city. Since then, thousands of cases have been reported with high lethality. In 2019, the lethality rate in the State of São Paulo was 9.25% [5], above the Brazilian average of 9.0% [6]. The Brazilian Visceral Leishmaniasis Surveillance and Control Program (VLSCP) focuses on reducing vectors, prompt human diagnosis and treatment, and controlling the main host, which includes the euthanasia of screened and confirmed seropositive dogs [7]. Canine treatment with Miltefosine, funded by the owners, is allowed [8], but it is not a coordinated public health measure. Despite these efforts, cases still have been reported in all Brazilian regions, and the disease is spreading to new areas.

In Brazil, the domestic dog is the main reservoir of *Leishmania infantum* in urban and peri-urban areas. This parasite is the agent of American Visceral Leishmaniasis (AVL) and is transmitted by the bites of female sand flies, mainly *Lutzomyia longipalpis, Lutzomyia cruzi* and possibly *Migonemyia migonei* [7,9].The parasite is recognized by its genomic plasticity and capability of rapid adaptation, ultimately altering its pathogenicity [10–12]. Recent studies demonstrated several *L. infantum* strains from Brazil presenting a large (>12 kb) genomic deletion involving four open reading frames of the tetrasomic chromosome 31 (chr31) that alters parasite biology and potentially the interplay with hosts [13,14]. It has been reported that the deletion-carrying strains (DEL) are more frequent and widely spread in Brazil [15]. However, to what extent such genotypes have spread within local areas in endemic regions is still unknown. Integrating genomic data into disease mapping could contribute to a better understanding of VL.

Several environmental drivers of VL have been identified in Brazil, including vegetation [16–18], and climatic factors, for instance, temperature, humidity, and precipitation [19,20]. These factors contribute to the formation of ecological niches of sand flies. Among them, vegetation is particularly important, as it can be easily and effectively identified using remote sensing techniques. Although there are various species of Phlebotominae, they all require vegetation, which provides nutrients and shaded environments for their survival. However, it remains unclear whether the presence of vegetation suffices or if the density, whether dense or sparse, affects disease transmission.

Human VL incidence has also been linked to the socioeconomic factors in the household microenvironment, such as living in slums, population density per household, poor living conditions, low levels of education or neighborhood literacy levels, households lacking sanitation and garbage collection [16,21–24]. Roads and transportation infrastructure have also been linked to the spread of VL [25], as the disease is often associated with rapidly expanding urban peripheries [23]. Despite these drivers, significant gaps remain in understanding CVL, particularly in local studies where there are small space variation. Few studies have focused on local-scale analyses to inform targeted control programs within small to medium-sized cities [26] and in large urban dog population [27] but still little is known about environmental and socioeconomic factors of CVL in local areas, using pixel scale of high-resolution images. Additionally, limited research has used Generalized Additive Models (GAMs) to predict VL [28] or its vectors [29]. Of note, GAMs have the ability to model complex environmental and socioeconomic factors and disease case occurrence, capturing spatial trends to understand the heterogeneous nature of VL transmission [28]. There is also a lack of understanding of the genotype dispersion processes of *L. infantum* in endemic areas and how this can influence the spatial distribution of disease cases.

The current study aims to i) identify and predict spatial patterns of CVL across an endemic urban area with a large (8,146) dog population sample, ii) map the genomic profile of the infecting *L. infantum* strains from dog samples collected across the city, and iii) relate the genetic profile to the observed spatial patterns of canine disease.

## 2. Materials and methods

### 2.1. Study site

**Fig 1:**
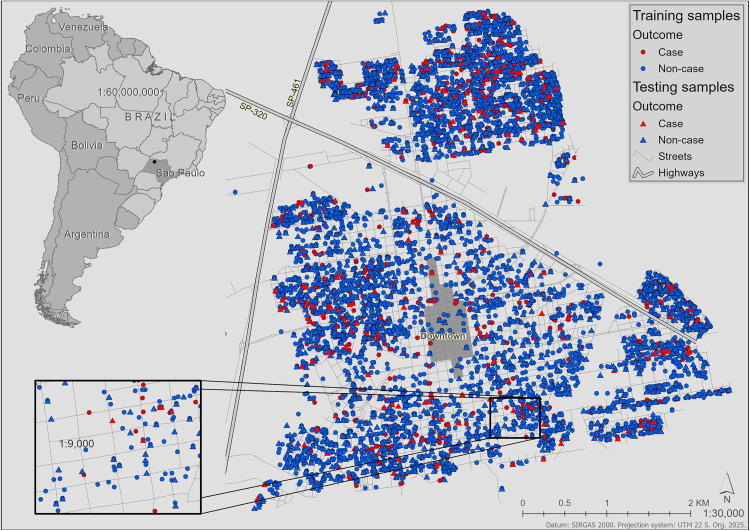
Distribution of canine samples stratified by diagnostic outcome. Circles represent the model training subset, and triangles the model testing subset. In the cartographic scale of 1:30,000, one dot may represent more than one dog sample due to the possibility of several sampled dogs in the same address. The source of South America boundaries is the GeoBoundaries [30]. Streets displayed in this figure were retrieved from OpenStreetMap and the OpenStreetMap Foundation, and are made available under the Open Database License.

Votuporanga is located in the northwest part of the State of São Paulo (20°25’22“S; 49°58’22”W – Fig 1). The municipality covers an area of 422.9 km^2^ and is traversed by one of the most important highways in the State, the SP-320 (Euclides da Cunha Highway), which gives access to the State of Mato Grosso do Sul (MS) and to the capital of the State of São Paulo (SP), also named São Paulo.

The population of Votuporanga was estimated at 100,159 inhabitants in 2024 [31]. The human development index was 0.790 in 2010 – similar to the São Paulo state average of 0.783 [32] but higher than the Brazilian average of 0.727 [33]. The canine population is estimated to be 25,088 dogs, using a study on the estimation of domestic animals in São Paulo State [34].

Votuporanga is located at 525 m of altitude and has an *Aw* equatorial climate with a dry winter, following the updated Köppen-Geiger climate classification system [35]. The annual mean temperature is above 22°C, with annual mean precipitation of approximately 1200 mm [36]. The biome of Votuporanga is Cerrado (tropical savanna) [32], and the municipality is located in the Planalto Ocidental Paulista plateau [37]. Native trees, such as *Bauhinia forficata* and *Moquilea tomentosa*, are frequently seen in the city’s urban landscape. Votuporanga has been one of the most at-risk municipalities for VL in São Paulo State, with *L. longipalpis* first reported in 2009, followed by canine disease cases in 2010 [38] and human cases in 2011 [5].

### 2.2. Ethical considerations

This research was performed with dogs whose owners voluntarily agreed to participate in the study under approval of the Brazilian ethical review Plataforma Brasil CAAE 63861716.2.0000.5402 – São Paulo State University – UNESP. Experimental procedures were approved by the Ethics Committee on Animal Experimentation of the Instituto Adolfo Lutz (CEUA nº. 01/2016) and by the School of Veterinary Medicine and Animal Science of the University of São Paulo (CEUA nº. 4079280115).

### 2.3. Study design and serosurvey

The study was open to all dog owners who wished to participate, and the serosurvey was conducted in communal locations, i.e., schools, gymnasiums, squares, and army reserve training locations, strategically localized to maximize community participation. The serosurvey was conducted from February 1 to September 26, 2014, and was originally part of a research project that aimed to investigate the effects of insecticide collars against CVL [39].

A total of 8,146 dogs was initially screened (S1 File, Fig S1) by the Dual Path Platform rapid test (TR-DPP®, Bio-Manguinhos/ FIOCRUZ), and seropositive results were confirmed by Enzyme-Linked Immunosorbent Assay (ELISA) (Bio-Manguinhos/ FIOCRUZ), as recommended by the Brazilian Ministry of Health [7]. The TR-DPP® is a specific test for *L. infantum* based on the reaction of IgG to the antigen rK28, performed according to the manufacturer’s recommendations. Seropositive dogs on both rapid test and ELISA were considered cases, while seronegative dogs in the rapid test were considered non-cases in the models. In this study, diagnoses were provided by the Center for Zoonoses Control, supervised by the Adolfo Lutz Institute, and funded by the Brazilian Ministry of Health.

### 2.4. *Leishmania infantum* detection and genotyping from infected dogs’ samples

Total DNA from 29 dogs was analyzed from approximately 30 mg of popliteal lymph node fragments, extracted using Illustra™ tissue and cells genomic Prep Mini Spin Kits (GE Healthcare Europe GmbH, Glattbrugg Switzerland) according to the manufacturer’s instructions. DNA pellets were dissolved in ultra-pure water. *L. infantum* infection in dog samples was confirmed by conventional PCR through the amplification of a 145-bp fragment of the variable region of the minicircle kDNA, according to Lachaud et al., 2002 [40]. For detecting the infecting parasite genotype, two pairs of primers were designed for relative quantitative PCR (qPCR) to amplify a deletion site on LinJ.31.2380 locus (a putative site responsible for encoding for 3’ NT/NU) and a downstream region on chromosome 31 (LinJ.31.2330), used as a constitutive reference site. The set of primers 5′-ACGATCGGCCTCAAAACACT-3′ and 5′- GGTGAAGTCTTCGTCCGTGT-3′ was designed to target LinJ.31.2380, within the chr31 deletion site; and primer sequences 5′-CGAACCTTGGAGCTTCCCTT-3′ and 5′-TCAAGGTTGTGTCCGTCGAG-3′ were designed to target LinJ.31.2330; downstream of the chr31 deletion site. DNA of the previously characterized non-deleted (Non-DEL) cultured strain IOCL 2666 was used as a reference for the relative quantification of the mutation, according to [15]. A pair of primers targeting canine DNA (HPRT – Hypoxanthine-guanine phosphoribosyltransferase) was included as an internal control for sample quality and amplification reaction. The standard protocol uses 0.2 nM of primers, 1x Sybr Green Master Mix, and 40 cycles at 62°C as the annealing temperature in an Applied Biosystems® 7500 Real-Time PCR System. Experiments were performed in triplicate techniques in two experimental replicates. The samples were classified as DEL (deletion-carrying sample) when the LinJ.31.2380 locus in chromosome 31 was not amplified, and as Non-DEL when both amplification in the LinJ.31.2330 and LinJ.31.2380 loci occurred with the same Ct values. When Ct deviation was observed, heterozygosity or a possible mixed population of parasites was assumed, samples were classified as HTZ/MIX.

### 2.5. Preparing datasets for spatial analysis

Dog addresses were geocoded using Google Fusion Tables (Google) based on the information provided by the dogs’ owners during the serosurvey. This technique converted addresses into latitude and longitude coordinates, which were then added to a Geographical Information System (GIS). We employed a combination of Google and Q-GIS 3.16, which successfully geocoded and mapped 8,146 canines (100%) locations. To ensure the accuracy of point placement, all points were manually verified and edited in ArcGIS Pro 3.1.2 (ESRI, Redlands, CA) as needed. Although 100% of the addresses were geocoded, approximately 10% of the points required manual adjustment due to mismatches in the automated geocoding process.

Vegetation cover was assessed using normalized difference vegetation index (NDVI) in Q-GIS 3.16 using RapidEye images (spatial resolution of 5 m) for August 2014, provided by the Brazilian Ministry of Environment [41]. NDVI was calculated according to equation 1 [42]:


NDVI = (NIR−Red)/(NIR+Red) (1)

Where NIR (near-infrared) corresponds to the spectral band wavelength range of 760 nm to 850 nm and the Red of 630 nm to 685 nm. NDVI varies by pixel from −1–1. Sparse vegetation, e.g., shrubs, grasslands, or senescing crops, usually results in a moderate NDVI, ranging from 0.2 to 0.5, while higher than 0.5 corresponds to dense vegetation, for instance, forests or crops at their peak growth stage [43]. We considered locations in the range of −0.69 to 0.2 as non-vegetated, ≥ 0.2 to 0.5 as sparse vegetation, and ≥ 0.5 as dense vegetation.

We created concentric buffers with a bandwidth of 100 meters around each dog’s address, considering the home range of pet dogs – which typically spend most of their time within a diameter of less than 250 meters around their owners’ homes [44], the short flight range of sand flies [45], and the typical length of Votuporanga’s streets, which is approximately 100m. Within these buffers, we calculated the mean NDVI, the number of vegetation patches, the number of patches of sparse vegetation, the number of patches of dense vegetation, the area of vegetation, the area of sparse vegetation, the area of dense vegetation, the number of buildings, the building area, and the mean street density (Table 1, S1 File, Fig S2).

**Table 1:** Initial variables for the construction of the models.

	Variable	Data Source
V1	Area of vegetation (m^2^)	NDVI, RapidEye Image
V2	Area of sparse vegetation (m^2^)	NDVI, RapidEye Image
V3	Area of dense vegetation (m^2^)	NDVI, RapidEye Image
V4	Number of vegetation patches	NDVI, RapidEye Image
V5	Number of sparse patches	NDVI, RapidEye Image
V6	Number of dense patches	NDVI, RapidEye Image
V7	NDVI	NDVI, RapidEye Image
V8	Mean NDVI	NDVI, RapidEye Image
V9	Median NDVI	NDVI, RapidEye Image
V10	Number of buildings	Vector data, Votuporanga City Hall
V11	Building area (m^2^)	Vector data, Votuporanga City Hall
V12	Street density	Vector data, DataGeo: Secretariat of Infrastructure and Environment of the State of São Paulo.

Abbreviations: NDVI, Normalized Difference Vegetation Index.

The variables were extracted from the NDVI raster file using spatial analysis tools in ArcGIS Pro 3.1.2 as well as from vector data provided by São Paulo State government [46] (i.e., streets) and the City Hall of Votuporanga (i.e., buildings). Street density was standardized ((Street density – mean)/ stddev) to aid identifiability of the effect estimate. Finally, the genomic profile of *L. infantum* parasites was analyzed from 29 euthanized and necropsied dogs and mapped.

### 2.6. Spatial clustering

Clustering of CVL cases was analyzed in the `splancs` package within the RStudio (Version 4.1.2), using Ripley’s K-function, as described by Chetwynd et al [47,48]. Here, *K(t)* is the number of events within a distance of an arbitrary event, divided by the overall density of events. Plotted envelopes show the maximum and minimum simulated values of *K*_*1*_*(t)* and *K*_*2*_*(t)* for cases and non-cases, respectively, at each separating distance. For significance testing using Monte Carlo simulation, we undertook 199 simulations, giving a significance threshold of *p = *0.05 for rejecting the null hypothesis.

### 2.7. Kernel density

The density of CVL cases was mapped using kernel density estimation [49], which calculates density based on the number of events per unit of area [50]. We estimated density according to equation 2:


$${\hat{\lambda }}_{\tau }(\text{s})=\sum_{d_{i}\leq \tau }\frac{3}{\pi \tau^{2}}{\left(1-\frac{d_{i}^{2}}{\tau^{2}}\right)}^{2}\mathrm{\backslash ]}$$ (2)

where, $d_{i}$ measures the distance between s and the event in location, $s_{i}$ and τ  is the radius centered on s. At a distance of zero, the weight is 3/$\pi \tau^{2}$ and drops smoothly to zero at distance τ [49]. Using ArcGIS Pro 3.1.2, we calculated the density of all canine samples, CVL cases specifically (S1 File, S3 Fig), and then created a kernel density ratio map (CVL: all samples) to provide a visual representation of CVL risk.

### 2.8. Logistic regression models and spatial prediction

Candidate variables (area of vegetation, area of sparse vegetation, area of dense vegetation, number of patches, number of patches of sparse vegetation, number of patches of dense vegetation, NDVI, mean NDVI, median NDVI, number of buildings, building area, and density of streets) were tested for multicollinearity in R-Studio 4.4.0 using the package ‘car’. We conducted a Variance Inflation Factors (VIF) test, initially excluding V1 (area of vegetation), V4 (number of vegetation patches) and V9 (median NDVI) due to high correlation. All remaining variables presented values < 5 (S1 File, Table S1). We also performed a correlation matrix for several pairs of variables, excluding those with a correlation coefficient > 0.70. i.e., V11 in addition to the previously identified variables (S1 File, Table S2). Next, we ran binary logistic regression models using the packages ‘questionr’, in which the outcome (CVL) was considered 0 (zero) for non-cases and 1 for cases.

Since mean NDVI, sparse vegetation, and street density were significantly associated with CVL (threshold of p < 0.05), we selected these covariates to run the spatial prediction model, which used a Generalized Additive Model (GAM) framework (S2 File). We used an approach reported for case-control data [48,51] and considered three spatial models according to the equations:


$$\text{logit}\left(P_{i}\right)={a+b\;\times NDVI}_{i}{\;+\;c\times \text{spar}}_{i}+{\;d\times \text{streets}}_{i}+\;{\text{S}}_{i}$$ (3)

$$\text{logit}\left(P_{i}\right)={a+b\;\times NDVI}_{i}+{\;d\times \text{streets}}_{i}+\;{\text{S}}_{i}$$ (4)

$$\text{logit}\left(P_{i}\right)={a+b\;\times NDVI}_{i}+\;{\text{S}}_{i}$$ (5)

where ${\text{Y}}_{i}=1$ for cases and ${\text{Y}}_{i}=0$ for non-cases (treated in the model as controls), NDVI is the mean NDVI value around location i, spar is the area of sparse vegetation around location i, streets is the street density around location i, a is the ratio of cases to non-cases, b represents the coefficient for NDVI, c the coefficient for area of sparse vegetation, d the coefficient for streets density, and ${\text{S}}_{i}$ is the residual spatial variation function after accounting for the effect of the covariates. ${\text{S}}_{i}$ was modeled using a locally estimated scatterplot smoothing (LOESS) regression smoother against the X and Y UTM coordinates (Datum SIRGAS 2000 UTM 22S).

We tested multiple bandwidths for the smoother and selected the optimal for the model based on Akaike’s Information Criterion (AIC) [48]. The first model (equation 3) used a span of 0.05, while the second (equation 4) and third (equation 5) used a span of 0.1. The models predicted adjusted log odds for each location on a prediction grid of 50m x 50m with raw NDVI, sparse vegetation area, numbers of households or vegetation density within a 100m radius at the location of intersecting grid points (S1 File, Fig S2). We then created a null model omitting the covariates and smoothing terms to provide a reference for calculating odds ratios at each location. The models were created in R-Studio using the `gam` package.

### 2.9. Cross-validation

We selected random points from canine samples in ArcGIS Pro 3.1.2, splitting our database into 75% (6,109 sampled dogs) for training and the remaining 25% (2,037 sampled dogs) for testing. Next, we used the model built on the training dataset to predict the odds of disease cases for the 2,037 testing locations for cross-validation. We then calculated the models’ sensitivity, specificity, and accuracy for correctly predicting the observed value of infection status (case or non-case) at the testing locations with a predicted threshold of spatial OR=1.0. The area under the curve (AUC) of the receiver operating characteristic (ROC) was calculated with 95% confidence intervals to determine the models’ predictive performance across all prediction value thresholds. The ROC values were interpreted according to the ranges of 0.5–0.6 as poor, > 0.6–0.7 as moderate, > 0.7–0.8 as good, > 0.8–09 as strong, and >0.9 as indicative of near-perfect performance, assuming 0.5 has no discrimination, with the ROC curve lying the major diagonal (true and false positive are equal) and, in contrast, 1.0 shows perfect discrimination, where the curve follows the left and upper borders, representing a true-positive proportion (1.0) for all values of the false-positive proportion [52]. The final spatial predictions were undertaken using the model built on the training dataset only. The cross-validation was conducted using the ‘pROC’ package and graphs were ploted using ‘ggplot2’ in R-Studio.

## 3. Results

From 8,146 canine samples examined, 1,066 (13%) tested seropositive (cases), while 7,080 (87%) tested seronegative (non-cases). Approximately 89% (947/1,066) of the positive dogs were euthanized according to the Public Health controlling recommendations, of which 66% (708/1,066) were euthanatized between February and December 2014, and the remaining 22% (239/1,066) between 2015 and 2017. Necropsy was performed on 29 euthanized dogs. They were all positive for *L. infantum*, of which, in the genotype analysis, 12 (41%) were HTZ/MIX, 10 (35%) Non-DEL, and seven (24%) DEL.

**Fig 2:**
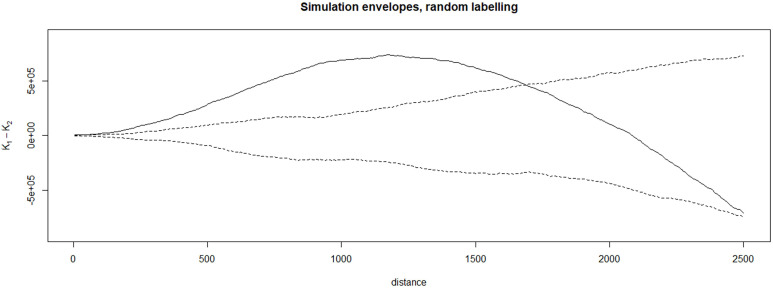
Degree of spatial clustering of CVL cases relative to the non-cases at different spatial scales. Upper and lower simulation envelopes are the dashed lines assuming no spatial clustering. The continuous black curve above the upper line of the envelope demonstrates positive, significant spatial dependency between cases relative to non-cases. Distance is in meters.

The K-function demonstrated significant spatial clustering of canine cases relative to non-cases up to approximately 1.7 km, with a peak at 1.2 km (Fig 2).

**Fig 3:**
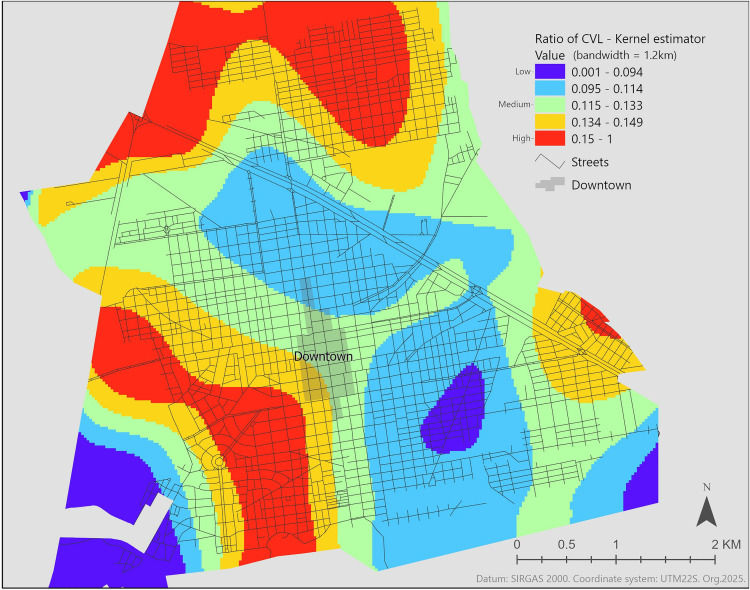
Kernel density ratio map for CVL leishmaniasis. Dark blue represents very low concentration of CVL risk, green medium, and red high. The method chosen for the classification of the histogram was Quantile. Abbreviations: CVL, canine visceral leishmaniasis. Streets displayed in this figure were retrieved from OpenStreetMap and the OpenStreetMap Foundation, and are made available under the Open Database License.

Kernel density maps were created with a bandwidth of 1,200 m, consistent with the observed peak of spatial clustering of the canine disease cases. The highest density of both cases and the total sampled population (S1 File, Fig S3, A and B, respectively) occurred in the north of the city. However, when analyzing only the concentration of positive cases, higher concentrations can be seen not only in the north but also extending from the west to the southwest and in the east (S1 File, Fig S3, A). This concentration might be influenced by the total number of samples collected, as illustrated in S1 File, Fig S3, B. To account for this, we calculated a density ratio for cases to all samples, which serves as an indicator of CVL risk. This ratio reveals higher concentrations of CVL risk in extensive areas in the north, southwest/south, and a small area in the east (Fig 3), all located on the fringes of the urban area.

Among all variables analyzed (i.e., area of sparse vegetation, area of dense vegetation, number of sparse vegetation patches, number of dense vegetation patches, mean NDVI, number of buildings, and street density), only the area of sparse vegetation (V2), mean NDVI (V8) and street density (V12) were statistically significant in the binary logistic regression model, with mean NDVI showing the highest odds ratio (Table 2).

**Table 2:** Logistic regression model for canine visceral leishmaniasis.

	OR	2.5%	97.5%
(Intercept)	0.127199***	0.085048	0.1886
V2 (area of sparse vegetation)	1.000034*	0.999994	1.0001
V3 (area of dense vegetation)	0.999992	0.999928	1.0001
V5 (number of sparse vegetation patches)	0.995898	0.982673	1.0093
V6 (number of dense vegetation patches)	0.995017	0.947120	1.0441
V8 (mean NDVI)	4.313393***	1.937254	9.5557
V10 (number of buildings)	1.000983	0.998077	1.0039
V12 (street density)	1.090468**	1.004621	1.1849

Significance codes: 0 ‘***’, 0.01 ‘**’, and 0.05’*’. Abbreviation: NDVI, Normalized Difference Vegetation Index.

**Fig 4:**
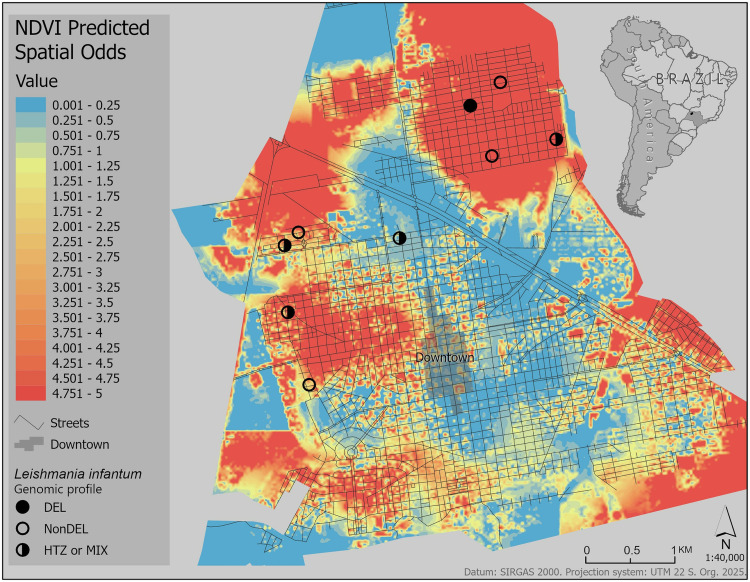
Spatial prediction of CVL risk derived from a GAM with mean NDVI as covariates. The blue color represents lower spatial odds, going to yellow for medium and red for higher. Del, non-DEL, HTZ/MIX isolates are distributed along the city. The source of South America boundaries is the GeoBoundaries [30]. Streets displayed in this figure were retrieved from OpenStreetMap and the OpenStreetMap Foundation, and are made available under the Open Database License. Abbreviations: GAM, Generalized Additive Model; NDVI, Normalized Difference Vegetation Index. DEL, deletion-carrying sample; HTZ/MIX, heterozygosity or a possible mixed population of parasites.

The three aforementioned significant covariates were all statistically significant in the spatial prediction models (Table 3) and showed moderate to good performance, with AUC values ranging from 0.64 to 0.74. Model 3 exhibited the best predictive ability, and its spatial predictions are presented in Fig 4.

**Table 3:** Performance of Generalized additive models (GAM) for canine visceral leishmaniasis.

	Model 1* NDVI, spar, streets	Model 2*** NDVI, streets	Model 3*** NDVI
AUC	0.64	0.65	0.74
CI	0.61 - 0.66	0.63 - 0.68	0.72 - 0.76
Sp	0.70	0.64	0.85
Se	0.51	0.62	0.54
Ac	0.61	0.63	0.65

Significance codes: 0 ‘***’ and 0.05’*’. Abbreviations: AUC, Area Under the Curve; CI, Confidence Interval; Sp, Specificity; Se, Sensitivity; Ac, Accuracy.

The kernel density ratio map shows consistencies with the GAM prediction map, particularly in the areas of low spatial odds to the east of the downtown area and high in the north and west of the city. In fact, nearly 45% of the city is in areas of increased odds for CVL. The majority of high spatial odds (>4) were in the periurban areas. In this regard, we found distinct genotypes of the dogs infected with *L. infantum* in different city areas; however, a DEL strain was found in a high-spatial-odds predicted area. Locations close to the downtown area, in the city center, were consistently found to have low spatial odds across all spatial analytical approaches.

**Fig 5:**
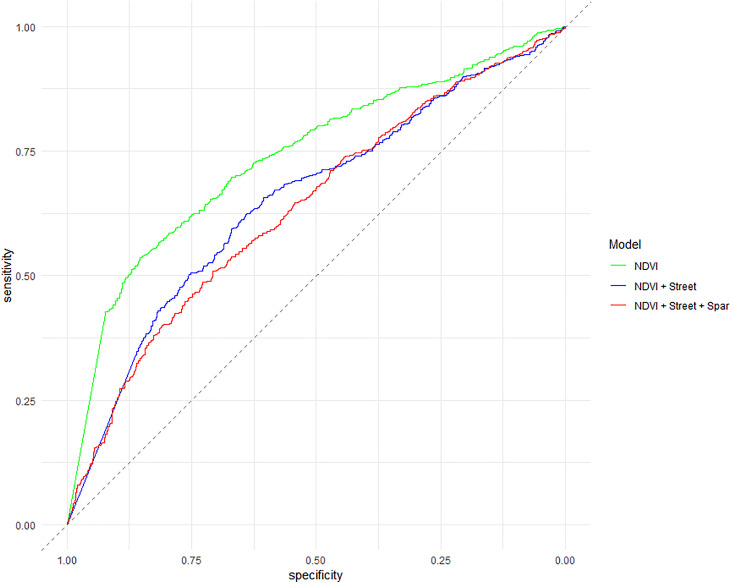
Receiver operating characteristic of predicted spatial odds from generalized additive models of CVL. Abbreviations: NDVI, Normalized Difference Vegetation Index, Streets, street density; Spar, area of sparse vegetation.

Overall, the models’ AUC values were moderate in predicting CVL disease status but the best performance was achieved by the model that used only NDVI (Fig 5), with an AUC of 0.74 and 95% confidence intervals between 0.72 and 0.76, indicating good predictive performance.

## 4. Discussion

This study identified a high seropositive rate among dogs (13%), which is consistent with the epidemiological profile of Votuporanga, a city with a known history of canine and human case records [5,38]. Even higher prevalence has been found in other Brazilian cities with similar characteristics, such as Aracatuba, where a canine seroprevalence of 21% was recorded between 2013 and 2022 [26].

Our study detected spatial clustering of CVL cases relative to non-cases within the boundaries of a medium-sized city in an endemic area of the northwest part of the State of São Paulo in southeastern Brazil, consistent with others’ findings of spatial clustering of visceral leishmaniasis at a range of spatial scales [53]. Clusters of CVL were approximately 1.7 km in diameter, highlighting significant small-scale spatial variation. Considering the importance of *L. longipalpis* in the transmission of VL and its flight range at short distances (50m-300m, with a maximum distance of 500m [45]), clusters are likely to consist of multiple overlapping communities of sand flies located within areas with suitable habitats for survival and breeding, including vegetation.

In an ecosystem like the one in Votuporanga, characterized by high temperatures and generally dry weather, vegetation plays a crucial role in the VL microenvironment [24]. It supports *L. longipalpis* survival as provides shaded environments with possible available organic matter, such as dry leaves, rotten logs, animal feces, and fruits, all working as suitable sand fly habitats. It provides shade, humidity, and organic detritus conducive to vector existence [54]. Female sand flies need blood from other animals but require vegetated areas to lay their eggs. Males, on the other hand, need only soft-stemmed edible plants for survival [55]. Both sexes of flies ultimately need vegetated areas to survive.

Despite these physiological requirements of sand flies, CVL cases have been reported in a variety of different environments, demonstrating a complex epidemiological pattern of VL. In Niterói, Rio de Janeiro, Brazil, CVL prevalence was fivefold higher in locations with less vegetation than in densely-vegetated areas [24,56]. In contrast, in Teresina, Brazil, neighborhoods with heavier vegetation had a higher incidence [23] and in Aracatuba, São Paulo, authors found an association between animals testing positive and higher vegetation density [26]. Another Brazilian study, in Teresina, Piaui, found that the odds of CVL were twice as high in households with lower vegetation cover than those with higher vegetation cover [57]. Highly vegetated areas were also associated with human cases disease in a meta-analysis study [24]. This suggests that local vegetation patterns are important for driving small-scale variation in sand fly population dynamics and disease transmission, emphasizing the importance of such studies.

We found that, individually, mean NDVI, street density, and sparse vegetation were significantly and positively associated with an increase of CVL outcome; however, the effect size of street density and sparse vegetation were relatively small. This may be related to the nature of the VL as a disease that, while urban, tends to concentrate in areas where vegetation more conducive to transmission. Although VL may occur in settings with sparse vegetation or along streets, its ecological dynamics may be more strongly influenced by the broader presence of vegetation rather than sparse vegetation specifically. Sparse vegetation, fragmented vegetation, and urban trees, such as those found in the urban landscape of Votuporanga (mostly *Moquilea tomentosa* and *Bauhinia forficata* species); however, might provide suitable conditions for *L. longipalpis* survival. A study in Rio de Janeiro found that areas with higher cover of sparse vegetation presented higher prevalence of CVL infection than those densely urbanized [56]. This study also found that more urbanized areas, such as those characterized by commercial buildings and gray areas were associated with lower occurrence of the disease. In agreement with these findings, our spatial model shows a lower association near downtown areas, characterized by urban infrastructure, multiple commercial buildings, and a lower presence of dense vegetation.

Although vegetation is crucial for sand flies’ survival and fits well in predicting CVL when considering both mean NDVI and sparse vegetation, the density of streets also indicates that anthropic factors play an important role in the epidemiological landscape of CVL. Initially, we thought that the density of streets could be negatively associated with CVL, as it differs from a natural sylvatic environment; however, street density was positively associated with CVL cases, possibly working as a proxy for households and canines, highlighting that vectors seem to be well adapted to the urbanized environment where high street density exists. Thus, places where dense vegetation or sylvatic environments are may no longer be as necessary as they used to be in the past. Indeed, our spatial model suggests that higher predicted spatial odds for CVL may indicate that the vectors are in close association with human beings and the microenvironment they inhabit, as highlighted by another study conducted in Mato Grosso do Sul, Brazil, which found a high frequency of sand flies in both vegetated and disturbed environments [58].

Our models predicted very high spatial odds in urban periphery areas, along the border of the urban and rural/sylvatic environments, a pattern similar to that found by Carvalho and et al. [59] and Neto et al. [23]. The urban fringe of this city has a dense population of dogs, identified by the concentration of samples examined, which can facilitate disease transmission both to other dogs and possibly to people [27]. In addition, although stray dogs were not included in this study, it should be noted that they are common in this area and play an important role in the perpetuation and spread of CVL with their errant behavior and high disease prevalence [60]. Successfully reducing CVL transmission in the northern periphery, where odds are the highest could minimize CVL odds across the entire city because the north might act as a reservoir of transmission. However, it is common to replace euthanized dogs in endemic Brazilian cities [61] and for there to be a regular entrance and exit of dogs in a family [62], potentially undermining the effectiveness of control strategies based on dog-culling [39,63]. Thus, removing infected dogs would need to be supported by ensuring new dogs do not become infected such as through insecticide collaring and other interventions, including environmental management, health education, and active surveillance with serological screening of dogs.

By exploring the genomic profile of the circulating *L. infantum* strains, we found different genotypes (DEL, Non-DEL, and HTZ/MIX) from the canine samples in distinct city areas. Previous studies show the parasites’ complex and diverse genomic profile in South America [15] and in São Paulo State [64,65]. It should be noted that Votuporanga is located in the northwest of the State of São Paulo, roughly 100 km from the Mato Grosso do Sul state border, easily connected by one highway, facilitating the movement of potentially infected dogs and thus producing a similar genomic profile among both states, as presented in Schwabl and authors [15]. Non-DEL *L. infantum* was typed in all Mato Grosso do Sul strains, while DEL was predominant in São Paulo State, mainly in the southwest [13,15,65]. Furthermore, we identified DEL strains in the north of the city. Studies showed the deletion of chromosome 31 (chr31) of *L. infantum* could be related to increased resistance to miltefosine treatment [13], and reduced ecto-3’-nucleotidase activity [15] might be associated with reduced ability to infect macrophages and low survival when in contact with neutrophil NET’s in vitro [66].

The circulation of different *L. infantum* genotypes and possible occurrences of coinfections inside the city suggest parasite presence might be associated with adaptation to different environments as well as population mixing and movement of infected dogs in and out of the city. *Leishmania* can rapidly adapt after an epidemic expansion [15]. Recombination among *L. infantum* populations and hybridization between divergent parasite isolates and other species can impact pathogenicity, virulence, tissue tropism, and drug resistance [10–12], enabling spread by the vectors [67] and increasing the risk of canine or human cases of VL. It is noteworthy that the use of Miltefosine to treat CVL could increase the dispersion of DEL parasites as they are reportedly less sensitive to treatment [13]. The dispersion of distinct genotypes of *L. infantum* within the cities is still unclear. Further investigation is thus needed, as the biology of vectors, reservoirs, environmental drivers such as vegetation cover, the socio-cultural habits of the dog owners, and public policies may influence the disease cycle and transmission.

Finally, the current investigation had limitations that should be acknowledged. A small number of samples were genotyped, and even fewer were geocoded. This was due to the need to perform necropsies to follow the standardization of the technique, which required frozen tissue fragments, veterinary teams, adequate room for necropsies, and the consent of the dog’s owner. To reach the limited sample size, we included samples of infected stray dogs scheduled for euthanasia. However, as stray dogs, we could not geo-locate these samples. Despite the restricted sampling, the genotyping revealed significant variability in the genomic profile of *L. infantum*. The geocoded cases presented a wide distribution across different parts of the city. Future genomic work with larger samples might allow for mapping the spatial distribution of genotypes and robust association testing with covariates.

Additionally, we constructed models using a limited set of candidate covariates focusing mainly on the environmental factors, as these were the only surfaces available at sufficient spatial resolution for our models. In the future, other covariates, such as temperature and population density (human, canine, and vector), might be added to the survey to enhance the understanding of the spatial epidemiology of CVL. Finally, as this study was embedded within a public health intervention involving a large number of sampled dogs, data collection was conducted by health agents rather than veterinarians. As a result, individual-level data such as breed, age, weight, health or vaccination status, behavior and other dog characteristics were not included in the models.

## 5. Conclusion

Mean NDVI, street density, and sparse vegetation were found to be good predictors of CVL outcomes, having mean NDVI the best performance. By applying spatial analysis, we identified areas with high spatial odds of CVL in nearly half of the city area, mainly on the outskirts of a mid-sized regional city in Brazil. Although vegetation plays a crucial role in maintaining environmental balance, promoting human health, and enhancing well-being, in VL-endemic areas, it can contribute to an increased odds of disease transmission. This highlights the need for targeted interventions, such as environmental management strategies and educational initiatives, to mitigate the spread of VL in these areas. We also detected a deletion-carrying *L. infantum* isolates in an area with high spatial odds of CVL. This underscores the importance of both environmental and anthropic covariates in disease distribution and demonstrates the potential for integrating biological data into mapping to enhance our understanding of disease transmission spatial dynamics.

## Supporting information

S1 File Methodological proceedings. Supplemental figures and tables.
(PDF)

S2 File Script and manuscript data. (TXT)
