# Supplementary material for: Spatial prediction of canine visceral leishmaniasis in an endemic urban area of Brazil
Source: PLoS One. 2025 Aug 29;20(8):e0330730. doi: 10.1371/journal.pone.0330730 (PMC12396649; doi:10.1371/journal.pone.0330730)
Supplement: S1 File — Supplemental figures and tables. (PDF) [file pone.0330730.s001.pdf]

Supporting Information I

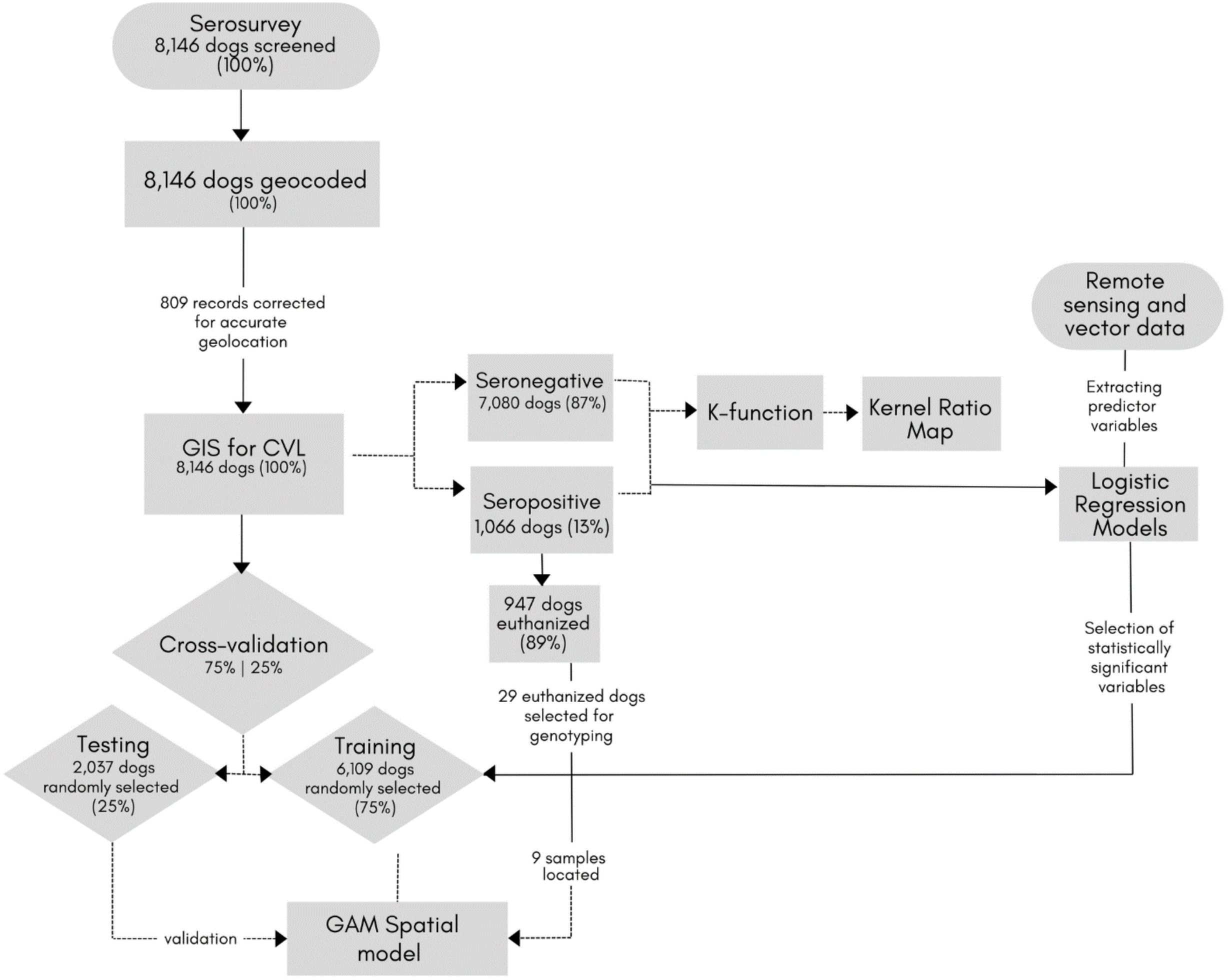

Fig S1. Methodological flowchart of sampling. A total of 8,146 dogs were initially screened for visceral leishmaniasis. All dogs were georeferenced, and the dataset was organized to support the K-function analysis, Kernel Ratio mapping, logistic regression modeling, cross-validation, and spatial prediction modeling. A subset of euthanized dogs was selected for genotyping, and some were located within the spatial model output. Lines represent the relationship of methodological steps, in which solid lines are non-spatial relationships, while dashed lines are spatial relationships.

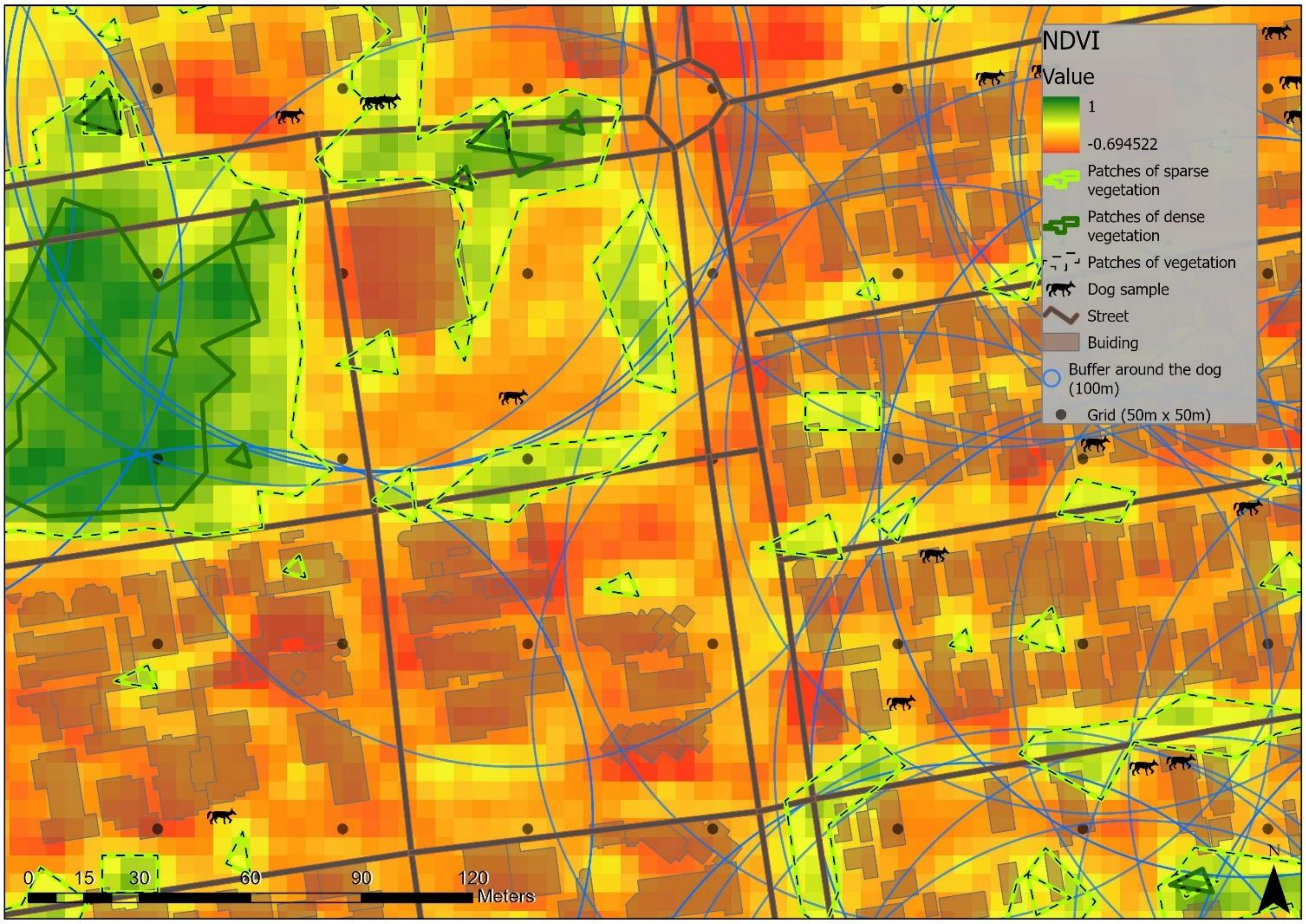

Fig S2. Creating the spatial dataset. Raster of NDVI and vegetation patches overlapped. A buffer of 100m was created around the dog sample and a 50m x 50 m grid was created to predict the GAM model based on the covariate values.

Table S1. Variance Inflation Factors to assess multicollinearity between variables.

Variable		VIF Value
V2	Area of sparse vegetation (m2)	3.24
V3	Area of dense vegetation (m2)	2.39
V5	Number of sparse patches	1.20
V6	Number of dense patches	1.42
V7	NDVI	1.10
V8	Mean NDVI	4.57
V10	Number of buildings	4.04
V11	Building area (m2)	4.27
V12	Street density	1.22

Abbreviation: VIF, Variance Inflation Factors.

Table S2. Correlation matrix used to assess multicollinearity between variables.

	V1	V2	V3	V4	V5	V6	V7	V8	V9	V10	V11	V12
V1	1.00	**0.89**	0.63	0.07	-0.07	0.49	0.17	**0.86**	**0.80**	-0.34	-0.40	-0.23
V2	**0.89**	1.00	0.21	0.19	0.05	0.51	0.18	0.70	0.69	-0.29	-0.33	-0.18
V3	0.63	0.21	1.00	-0.18	-0.24	0.18	0.05	0.63	0.54	-0.25	-0.30	-0.19
V4	0.07	0.19	-0.18	1.00	**0.96**	0.35	0.08	0.07	0.05	0.14	0.20	0.13
V5	-0.07	0.05	-0.24	**0.96**	1.00	0.08	0.08	-0.03	-0.03	0.22	0.28	0.18
V6	0.49	0.51	0.18	0.35	0.08	1.00	0.03	0.35	0.29	-0.25	-0.21	-0.14
V7	0.17	0.18	0.05	0.08	0.08	0.03	1.00	0.23	0.23	0.06	-0.03	0.04
V8	**0.86**	0.70	0.63	0.07	-0.03	0.35	0.23	1.00	**0.96**	-0.21	-0.34	-0.18
V9	**0.80**	0.69	0.54	0.05	-0.03	0.29	0.23	**0.96**	1.00	-0.23	-0.36	-0.17
V10	-0.34	-0.29	-0.25	0.14	0.22	-0.25	0.06	-0.21	-0.23	1.00	**0.85**	0.38
V11	-0.40	-0.33	-0.30	0.20	0.28	-0.21	-0.03	-0.34	-0.36	**0.85**	1.00	0.41
V12	-0.23	-0.18	-0.19	0.13	0.18	-0.14	0.04	-0.18	-0.17	0.38	0.41	1.00

Bold values indicate high correlations between variable pairs, which were excluded from the model. V1, Area of vegetation (m2); V4, Number of vegetation patches; V9, Median NDVI.

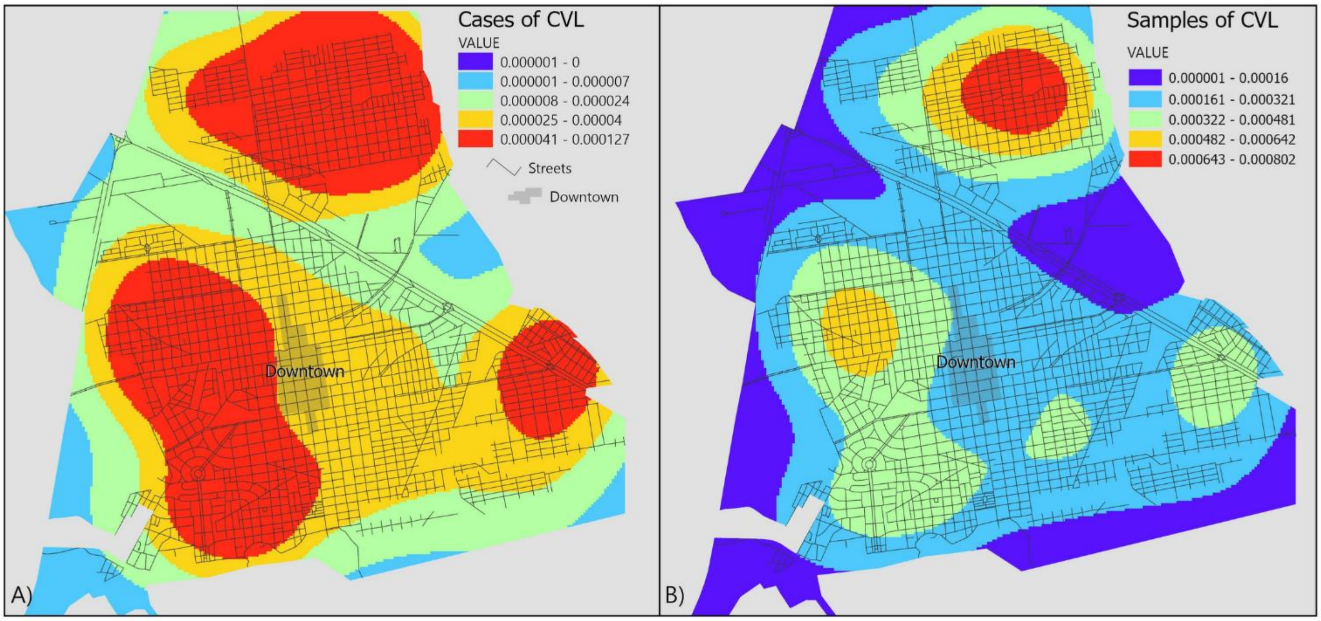

Fig S3. Kernel estimator maps. A) Kernel estimator map for cases of CVL B) Kernel estimator map for samples collected. The method of classification of the histogram was Quantile.
